# Next Generation Sequencing Identifies Five Novel Mutations in Lebanese Patients with Bardet–Biedl and Usher Syndromes

**DOI:** 10.3390/genes10121047

**Published:** 2019-12-16

**Authors:** Lama Jaffal, Wissam H Joumaa, Alexandre Assi, Charles Helou, George Cherfan, Kazem Zibara, Isabelle Audo, Christina Zeitz, Said El Shamieh

**Affiliations:** 1 Department of Biological and Environmental Sciences, Faculty of Science, Beirut Arab University, Debbieh 1107 2809, Lebanon; lama.jaffal66@gmail.com; 2 Rammal Hassan Rammal Research Laboratory, Physiotoxicity (PhyTox), Faculty of Sciences, Lebanese University, Nabatieh 1700, Lebanon; wjoumaa@ul.edu.lb; 3 Retinal Service, Beirut Eye & ENT Specialist Hospital, Beirut 1106, Lebanon; alexassi@hotmail.com (A.A.); charleshelou@hotmail.com (C.H.); georgecherfan@gmail.com (G.C.); 4 ER045, PRASE, DSST, Lebanese University, Beirut 1700, Lebanon; kzibara@ul.edu.lb; 5 Biology Department, Faculty of Sciences-I, Lebanese University, Beirut 1700, Lebanon; 6 Sorbonne Université, INSERM, CNRS, Institut de la Vision, 75012 Paris, France; isabelle.audo@inserm.fr (I.A.); christina.zeitz@inserm.fr (C.Z.); 7 CHNO des Quinze-Vingts, INSERM-DGOS CIC1423, 75012 Paris, France; 8 University College London Institute of Ophthalmology, London EC1V 9EL, UK; 9 Department of Medical Laboratory Technology, Faculty of Health Sciences, Beirut Arab University, Beirut 1107 2809, Lebanon

**Keywords:** inherited retinal diseases, next generation sequencing, mutations, Sanger sequencing

## Abstract

Aim: To identify disease-causing mutations in four Lebanese families: three families with Bardet–Biedl and one family with Usher syndrome (BBS and USH respectively), using next generation sequencing (NGS). Methods: We applied targeted NGS in two families and whole exome sequencing (WES) in two other families. Pathogenicity of candidate mutations was evaluated according to frequency, conservation, in silico prediction tools, segregation with disease, and compatibility with inheritance pattern. The presence of pathogenic variants was confirmed via Sanger sequencing followed by segregation analysis. Results: Most likely disease-causing mutations were identified in all included patients. In BBS patients, we found (M1): c.2258A > T, p. (Glu753Val) in *BBS9*, (M2): c.68T > C; p. (Leu23Pro) in *ARL6*, (M3): c.265_266delTT; p. (Leu89Valfs*11) and (M4): c.880T > G; p. (Tyr294Asp) in *BBS12*. A previously known variant (M5): c.551A > G; p. (Asp184Ser) was also detected in *BBS5*. In the USH patient, we found (M6): c.188A > C, p. (Tyr63Ser) in *CLRN1*. M2, M3, M4, and M6 were novel. All of the candidate mutations were shown to be likely disease-causing through our bioinformatic analysis. They also segregated with the corresponding phenotype in available family members. Conclusion: This study expanded the mutational spectrum and showed the genetic diversity of BBS and USH. It also spotlighted the efficiency of NGS techniques in revealing mutations underlying clinically and genetically heterogeneous disorders.

## 1. Introduction

Inherited retinal diseases (IRD) are a large group of clinically and genetically heterogeneous disorders characterized by loss of function of retinal photoreceptors, eventually leading to blindness [1]. Altogether, they affect approximately 1 in 3000 people [2,3], and can be inherited according to any Mendelian inheritance pattern [1]. IRD constitute the leading cause of visual impairment and blindness, especially in communities where the rates of consanguinity are elevated, with a significant impact on patients’ daily life and engagement in society [4,5,6].

IRD may affect the retina alone (non-syndromic IRD) or may occur in conjunction with other systemic disorders (syndromic IRD). Among the non-syndromic forms, the most frequent subtype is retinitis pigmentosa (RP) also known as rod-cone dystrophy (RCD) [5] which affects primarily the rod photoreceptors responsible for dim light vision, and secondarily the cone photoreceptors responsible for day light and precise color vision [7]. On the other hand, the most common type of syndromic RP is Usher syndrome (USH) [8], combining visual impairment to various degrees of hearing loss [9]. Prevalence of USH varies among populations, ranging from 1 to 4 per 25,000 individuals [8]. Another form of syndromic RP is the pleiotropic Bardet–Biedl syndrome (BBS) where retinal dystrophy is associated with a range of systemic pathologies classified into major and minor features, such as truncal obesity, polycystic kidney disease that can evolve toward renal insufficiency, polydactyly, genital anomalies, and learning difficulties [10,11]. Prevalence of BBS is population-dependent with significant variations ranging from 1:100,000 in North America and Europe [12], to 1 in 65,000 in a mixed Arab population [13]. Much higher rates are found in certain isolated consanguineous communities such as Kuwaiti Bedouins and Newfoundland, where the incidence rates attain 1:13,500 and 1:18,000, respectively [14,15]. Although not yet reported, the incidence of IRD in the Lebanese population is expected to be higher than the worldwide estimations, due to the high rates of consanguinity [16].

According to the Retinal Information Network database (RetNet: https://sph.uth.edu/retnet/), more than 268 genes are related to IRD so far. In most cases, the causative gene cannot be predicted based on the phenotype due to clinical and genetic overlaps, adding further complexity [17], [18]. This makes accurate genetic diagnosis that relies on conventional techniques a laborious task. For instance, detecting IRD’s underlying mutations using Sanger sequencing is labor-intensive, highly expensive and time-consuming [1]. Since alternative methods efficiently overcome these obstacles and provide a reliable and rapid tool for the diagnosis of IRD, we applied targeted [19] and whole next generation sequencing (NGS) to reveal the underlying genetic defects leading to BBS and USH in five patients belonging to four Lebanese families.

## 2. Materials and Methods

### 2.1. Ethics Statement and Clinical Examinations

All procedures adhered to the tenets of the Declaration of Helsinki. The institutional review board of Beirut Arab University approved the study protocol under IRB code: 2017H-0030-HS-R-0208. Written informed consent was obtained from all patients who had a presumed clinical diagnosis with IRD at Beirut Eye and ENT Specialist Hospital (Beirut, Lebanon), where they underwent clinical ophthalmic examination, as previously described [20].

### 2.2. Molecular Analysis and Mutations Detection

#### 2.2.1. DNA Extraction

Whole blood samples were taken from patients and their available family members. Genomic DNA was extracted using a DNA extraction kit from Qiagen (QIAamp DNA Mini Kit, Hilden, Germany). The quantity of DNA was measured using a Qubit 3.0 fluorometer (Thermo Fisher Scientific, Shah Alam, Malaysia).

#### 2.2.2. Targeted Next-Generation Sequencing

DNA samples of indexes FA4: V.3 and FD10: III.3 were analyzed using targeted NGS. The panel was selected from the Sure Select Human All Exon Kits Version4 (Agilent, Massy, Les Ulis, France). This panel covers 198 known IRD genes reported in RetNet (https://sph.uth.edu/retnet/) and literature. The eArray web-based probe design tool that was used for this purpose may be accessed at https://earray.chem.agilent.com/earray. Sequences were captured, enriched and eluted according to Agilent’s instructions, as previously reported [19]. The overall sequencing coverage of the targeted regions was ≥88% for a 25× depth of coverage.

#### 2.2.3. Whole-Exome Sequencing

We performed whole exome sequencing (WES) for DNA samples of indexes FB22: II.1 and FC51: II.2. Exons were captured and enriched using Agilent Sure select version 6. Captured libraries were then sequenced on NovaSeq6000 sequencer (Illumina) as 150 bp paired-end reads, following the manufacturer’s protocols. The mean coverage was 200× on raw data and >100× on target. Bioinformatic analysis of raw sequencing data was achieved using the Genome Analysis Toolkit (GATKv3.4.0) [21]. The GATKv3.4.0 pipeline tool was used to align reads to the human reference sequence (UCSC Genome Browser hg19) and for calling and annotating sequence variants (single nucleotide variations and indels). The variant calling process was done for single nucleotide variations and for indels separately.

#### 2.2.4. Analysis of Annotated Sequencing Data

All common polymorphisms with a minor allele frequency (MAF) higher than 0.01 were filtered out using several public databases such as Ensembl GRCh37 genome browser [22], 1000 genomes database [23], database of single nucleotide polymorphisms (dbSNP build 152) (https://www.ncbi.nlm.nih.gov/snp/), exome aggregation consortium database (ExAC) [24], genome aggregation database (gnomAD) [25] and trans-omics for precision medicine (TOPMed) Program [26], as these were considered to be common variants. The next filtering step was based on annotation type where inframe insertions/deletions (InDels), intronic, synonymous and variations in untranslated regions were excluded. In contrast, nonsense, missense variations and frameshift InDels located in exons or splice sites were prioritized. Thereafter, we verified if the remaining variants were found in dbSNP and NCBI databases.

#### 2.2.5. In-Silico Evaluation of the Pathogenicity of Candidate Mutations

The genome browser of the University of California at Santa Cruz (UCSC) [27] was used to check if the substituted amino acid is evolutionary conserved across different species including primates and main placental mammals. Not conserved residues, especially in other mammals and primates, were excluded. The details were described elsewhere [28].To check the phenotype(s) associated with the genes in which candidate mutations were identified, and to validate if the described phenotype matches the clinical diagnosis of patients, the RetNet database (https://sph.uth.edu/retnet/) was used. *In-silico* programs including scale-invariant feature transform (SIFT) [29], PolyPhen-2 [30] and MutationTaster2 [31] were used to predict the possible impact of the detected amino acid substitutions.

#### 2.2.6. Polymerase Chain Reaction, Sanger Sequencing, and Co-Segregation Analysis

Putative pathogenic mutations identified by NGS were confirmed through conventional polymerase chain reaction (PCR) (T100, Biorad, Kaki Bukit, Singapore) followed by Sanger sequencing (Applied Biosystems 3730xl DNA Sequencer, Courtaboeuf, Les Ulis, France) to eliminate the possibility of false positives. Primers for candidate mutations were designed using Primer3 (v.0.4.0) [32] and are available upon demand. Furthermore, DNA samples from available family members were also Sanger sequenced in order to carry out familial co-segregation analysis, as previously described [33].

### 2.3. Genotype–Phenotype Associations

The novelty of a candidate variant or whether it was previously reported to cause IRD was ascertained using several databases such as Human Gene Mutation Database [34], Leiden Open Variation Database [35], PubMed (https://www.ncbi.nlm.nih.gov/pubmed/) and Online Mendelian Inheritance in Man (https://omim.org/).

## 3. Results

This study includes three families with four patients suffering from BBS, and one family with one patient suffering from USH. The ophthalmologic clinical findings of these patients are summarized in Table 1. It is to be noted that the clinical diagnosis of all the BBS patients is in line with the diagnostic criteria of Beales et al. [10], stating that either four major BBS features or at least three major features combined to two minor features must be present (Table 2). 

**Figure 1:**
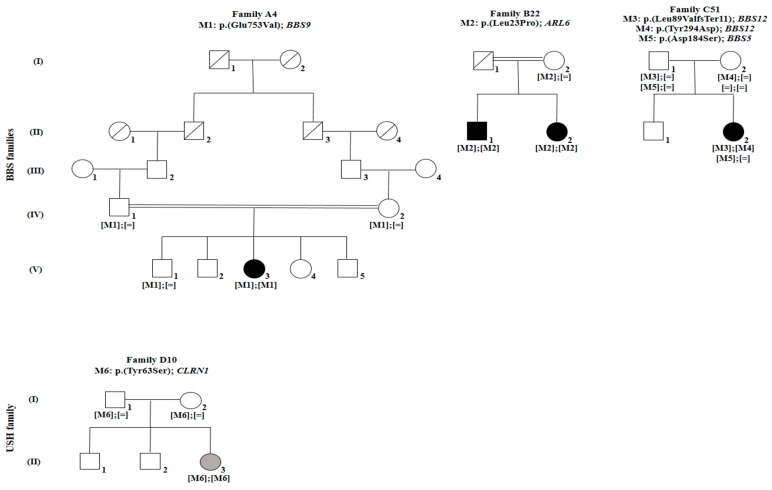
Pedigrees of three families with Bardet–Biedl syndrome (A4, B22, and C51) and one family with Usher syndrome (D10). White symbols indicate unaffected members. Black symbols indicate members affected with Bardet–Biedl syndrome. Gray symbols indicate members affected with Usher syndrome. Square and round symbols represent males and females, respectively. The slash indicates deceased individuals. Double horizontal lines represent consanguineous unions. M defines mutation.

**Figure 2:**
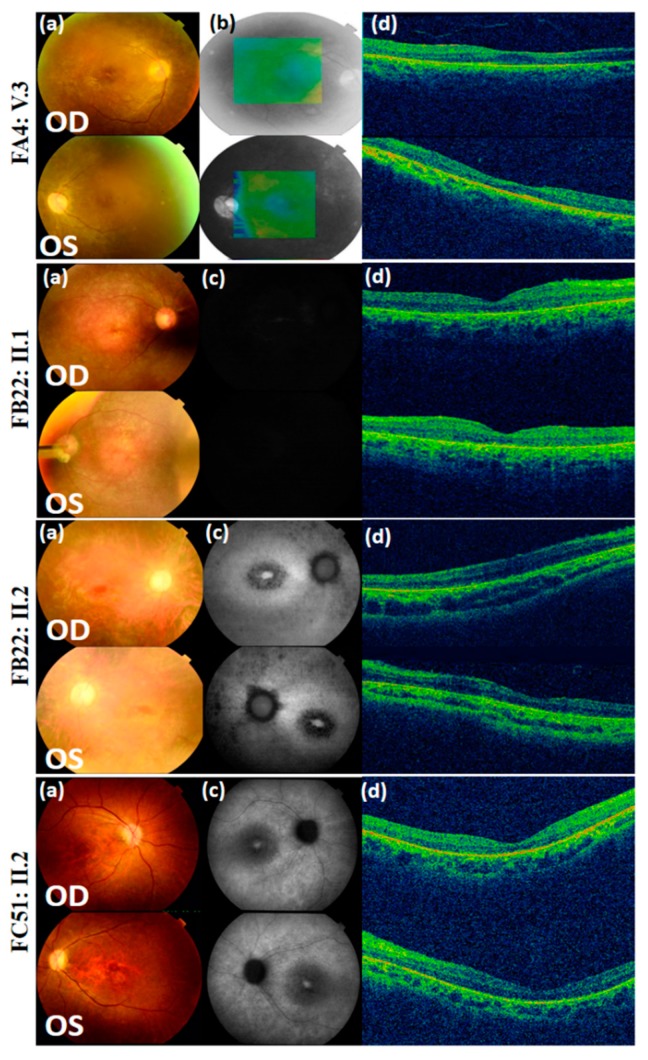
Color fundus photographs (**a**), red free fundus photographs (**b**), auto-fluorescence pictures (**c**), and optical coherence tomography (OCT) scans (**d**) of Bardet–Biedl syndrome patients FA4: V.3, FB22: II.1, FB22: II.2, and FD51: II.2. OD = oculus dexter; OS = oculus sinister.

In family A4 (FA4), index FA4: V.3 is a 24 year-old female diagnosed with BBS at 12 years, although she started experiencing reduced vision at the age of 6, with no family history. Her family presents a complex consanguinity case where the grand-fathers of her parents were brothers and hence her grand-fathers are first-degree cousins (Figure 1). Her fundus photographs showed peripheral pigmentary changes associated with macular involvement while the corresponding optical coherence tomography (OCT) showed bilateral retinal layer thinning at the macula (Figure 2). Moreover, she presented severely reduced scotopic and photopic full-field electroretinogram (ERG) responses. In addition to the visual impairment, she suffered from truncal obesity, learning difficulties, developmental delay, hypodontia and hepatic fibrosis (Table 2). NGS showed that she harbors a homozygous missense mutation (M1): c.2258A > T, p. (Glu753Val), rs61764068 in exon 20 of *Bardet–Biedl syndrome 9* gene (*BBS9*). Mutation M1 was shown to be rare and never homozygous in ExAC, gnomAD, and TOPMed populations (T = 0.0007685, 0.0007475, and 0.0006769 respectively, Table 3), affecting a highly conserved residue (Glu753) among different species according to the UCSC genome browser. It was also predicted to be probably damaging, deleterious and disease-causing according to PolyPhen-2, SIFT, and MutationTaster2, respectively. According to the standards developed by the American College of Medical Genetics and Genomics (ACMG) for the classification of sequence variants [36], M1 can be categorized as likely pathogenic (Table S1). This mutation has previously been reported in a compound heterozygous state with a second *BBS9* mutation, but in association with non-syndromic cone-rod dystrophy (CRD) [37]. The presence of M1 in index FA4: V.3 was validated using Sanger sequencing. Available unaffected family members (FA4: IV.1, IV.2, and V.1) were screened and were all found to be heterozygous for M1, indicating that this mutation co-segregated with BBS (Figure 1). The search for additional incidental mutation(s) in any BBS genes was done and showed no evidence for such mutation (Table S2).

In family B22 (FB22), indexes FB22: II.1 and FB22: II.2 correspond to a 34 year-old male and a 28 year-old female, with no family history, who were diagnosed with BBS. They both started having vision problems at 3 years, however, they were diagnosed at the ages of 5 and 3, respectively. Fundus examination for both indexes revealed bilateral widespread pigmentary changes outside the vascular arcades while OCT showed reduction of retinal thickness (Figure 2). They both presented severely reduced scotopic and photopic ERG responses (Table 1). In addition, FB22: II.1 suffered from truncal obesity, learning difficulties, developmental delay, hypodontia, cataract, brachydactyly, syndactyly, clinodactyly, and coordination problems. FB22: II.2 suffered from truncal obesity, polydactyly in the four limbs, learning difficulties, developmental delay, astigmatism, diabetes mellitus, brachydactyly, syndactyly, clinodactyly, and coordination problems (Table 2). Noting that learning difficulties and developmental delay were much more severe in FB22: II.1 than in his sister. Both patients carried the homozygous missense mutation (M2): c.68T > C; p. (Leu23Pro); rs1359075294 in exon 3 of the *ADP ribosylation factor like GTPase 6* gene (*ARL6*) also known as *Bardet–Biedl syndrome 3* (*BBS3*). No additional incidental mutation in any of the BBS genes was found leaving M2 as the only candidate mutation (Table S2). Mutation M2 was not detected in ExAC nor gnomAD populations while it was shown to be rare heterozygous in TOPMed (C = 0.0000079), affecting the well-conserved amino acid Leu23. Moreover, it was predicted to be probably damaging, deleterious, and disease-causing according to PolyPhen-2, SIFT and MutationTaster2; respectively. According to the ACMG standards [36], M2 can be categorized as likely pathogenic (Table S1). The presence of M2 in homozygous state was confirmed using Sanger sequencing in both affected members of this family, while it was heterozygous in their mother (Figure 1), confirming the co-segregation with the disease. M2 is also novel as it was not previously reported.

In family C51 (FC51), index FC51: II.2 is a 16 year-old female with no family history who was diagnosed with BBS at 13 years after starting complaining from reduced vision at the age of 11. Color fundus revealed atrophy at the macula in both eyes. Fundus autofluorescence images showed reduced autofluorescence at the maculae while OCT scans showed bilateral thinning of the neuroretinal layers at both maculae (Figure 2). Moreover, ERG responses were severely reduced for both the cone and rod systems. FC51: II.2 also suffered from truncal obesity, learning difficulties, speech delay, astigmatism, and clinodactyly (Table 2). This patient was shown to be compound heterozygous for two novel mutations in exon 2 of the *Bardet–Biedl syndrome 12* gene (*BBS12*). The first mutation is a frameshift deletion (M3): c.265_266delTT; p. (Leu89Valfs*11); rs1397714772, affecting a highly conserved residue and introducing a premature termination codon, 11 amino acids downstream. Furthermore, M3 was not detected in the ExAC population while it was found to be rare heterozygous in gnomAD and TOPMed populations (0.000012 and 0.0000079; respectively). According to the ACMG standards [36], M3 can be categorized as pathogenic (Table S1). However, the second mutation is a missense mutation (M4): c.880T > G; p. (Tyr294Asp). This Tyr294 residue is highly conserved in primates and marginally conserved in other mammals, but the missense variation into Asp was not found in any species. Prediction tools did not anticipate a deleterious effect but the mutation is extremely rare as it was never found in any of the ExAC, gnomAD, nor TOPMed populations (Table 3). According to the ACMG standards [36], M4 can be categorized as likely pathogenic (Table S1). Using Sanger sequencing, both M3 and M4 were confirmed to be present in index FC51: II.2. In addition, both also segregated with the disease as the father (FC51: I.1) was heterozygous for M3 and did not carry M4, while the mother (FC51: I.2) did not carry M3 and was heterozygous for M4 (Figure 1). On the other hand, index FC51: II.2 was also shown to carry a third heterozygous missense variant (M5) in exon 2 of the *Bardet–Biedl syndrome 5* gene (*BBS5*): c.551A > G; p. (Asp184Ser); rs137853921. The father (FC51: I.1) was heterozygous for this variant while it was absent in the mother (FC51: I.2). M5 had good predictions when assessed via in silico tools, but it had relatively high frequencies and was detected in a homozygous state in several individuals of the ExAC, gnomAD, and TOPMed general populations (G = 0.004381, 0.00416, and 0.0038 respectively, Table 3). According to the ACMG standards [36], M5 is considered a variant of uncertain significance (Table S1). Interestingly, M5 was previously reported as a potential modifier of the BBS phenotype [38].

**Figure 3:**
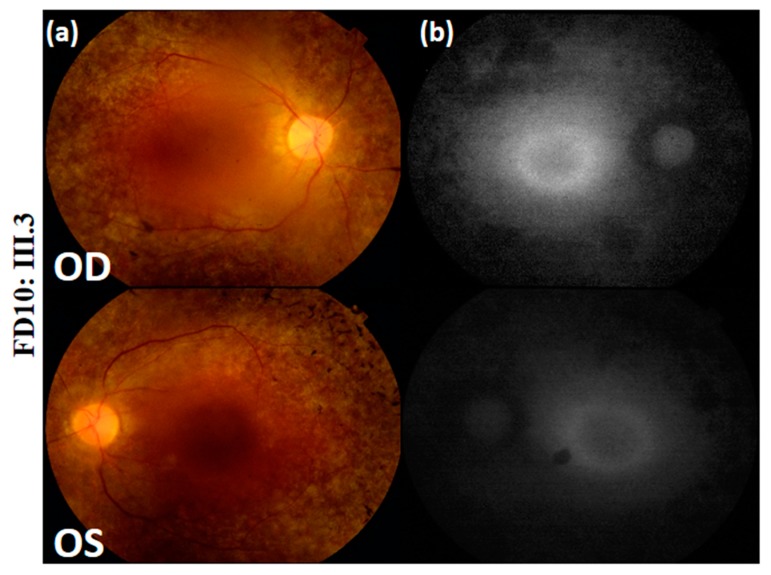
Color fundus photographs (**a**) and auto-fluorescence pictures (**b**) of Usher syndrome patient FD10: III.3. OD = oculus dexter; OS = oculus sinister.

In the fourth family D10 (FD10), index FD10: III.3 is a 40-year-old female who started experiencing reduced vision at 15 years, time at which she had an initial diagnosis of RCD. However, she was not diagnosed with USH until the age of 28, when the hearing loss began to appear. Her hearing problem was progressive and was accompanied with occasional dizziness, unsteadiness and loss of balance. Fundus photographs showed peripheral retinal pigment with central hyperautofluorescence on autofluorescence imaging (Figure 3). In addition, she presented severely reduced ERG responses under both, scotopic and photopic conditions. FD10: III.3 harbored the missense homozygous mutation (M6): c.188A > C, p. (Tyr63Ser) in exon 1 of *clarin 1* (*CLRN1*). No additional incidental mutation in any of the USH genes was found leaving M6 as the only candidate mutation (Table S2). It is important to note that M6 was never detected in any of the ExAC, gnomAD nor TOPMed populations. This mutation affected a highly conserved amino acid, and was predicted to be probably damaging, deleterious and disease-causing according to PolyPhen-2, SIFT and MutationTaster2; respectively (Table 3). According to the ACMG standards [36], M6 can be categorized as likely pathogenic (Table 1). It co-segregated with the disease as both parents were heterozygous carriers (Figure 1). This variation was not previously reported in the literature, making it novel.

## 4. Discussion

In the current study, NGS followed by extensive bioinformatic analysis revealed the presence of four novel and two known mutations in four Lebanese families. Four disease-causing mutations were associated with BBS; (M1): c.2258A > T, p. (Glu753Val) in *BBS9* [37], (M2): c.68T > C; p. (Leu23Pro) in *ARL6*, (M3): c.265_266delTT; p. (Leu89ValfsTer11) and (M4): c.880T > G; p. (Tyr294Asp) in *BBS12* while one disease-causing mutation was associated with USH; (M6): c.188A > C, p. (Tyr63Ser). In addition, one variant; (M5): c.551A > G; p. (Asp184Ser); rs137853921 that was previously reported as a possible phenotype modifier [38], was also found. All of the likely pathogenic mutations were validated by Sanger sequencing, co-segregated adequately with the corresponding phenotypes and showed an autosomal recessive mode of inheritance. Indeed, all of the available asymptomatic family members were either heterozygous carriers of the disease-causing mutations or carried the reference sequence (Figure 1).

The three BBS families carried likely pathogenic mutations in *BBS9, ARL6,* and *BBS12*. Previous studies showed that the majority of BBS cases in Northern Europe and North America are caused by mutations in *BBS1* and *BBS10* [39], none of them were found mutated in the Lebanese patients included in our study. Notably, the *ARL6* gene mutated in FB22, was reported to be unusually predominant in Saudi [40] and Indian [41] patients, in contrast to Caucasian patients in which *ARL6* mutations are extremely rare [42]. This could indicate the distinct prevalence of causative genes among different ethnicities, but this needs to be validated in larger cohorts.

To date, 21 genes (*BBS1-21*) are associated to BBS [43,44]. The BBS9 protein, altered in FA4, is part of a complex called the BBSome along with seven other BBS proteins. The latter complex is involved in primary cilium biogenesis, trafficking of proteins within cilia, as well as modulating intra-flagellar transport [45]. BBS9 is also a member of the BBSome core complex -along with BBS2 and BBS7- that plays an important role in the assembly of the whole mature BBSome [46]. This highlights the importance of BBS9 that was altered in FA4: V.3 by the homozygous missense mutation M1 replacing the conserved Glu753 residue, which is hydrophilic, polar, negatively charged, and acidic by a hydrophobic, non-polar, uncharged, and neutral Val residue. These deviations might possibly cause an impact on the solubility and the structure of the protein, consequently on its function. It is of particular interest, that M1 was previously found in a compound heterozygous state by Lee et al. in 2015, but in association with non-syndromic CRD [37], while in our study, it is associated with syndromic BBS. One possible explanation is that this mutation results in a more severe phenotype when both alleles are mutated, while a milder non-syndromic phenotype occurs when it is in combination with another *BBS9* mutation.

The *ARL6* gene, altered in FB22, encodes the ARL6 GTP-binding protein which is not part of the BBSome complex, but is responsible for many activities essential for the proper ciliary localization and trafficking of this complex: ARL6 was proposed to regulate the BBSome recruitment to the membranes, its coat-like assembly, its access to the ciliary compartment, in addition to its exit from the cilia [47,48]. The two FB22 patients were homozygous for the novel mutation M2; p. (Leu23Pro), that was rare heterozygous in TOPMed but absent in ExAC and gnomAD, and was predicted to be pathogenic in all prediction tools used in this study. Noteworthy, the phenotypic expressivity of BBS is not the same in the two affected siblings of this family (FB22: II.1 and FB22: II.2). For instance, learning difficulties and developmental delay were much more severe in patient II.1, whereas polydactyly in the four limbs was present in his sister II.2 only. More differences in the symptoms’ manifestations were found on the level of minor BBS features (Table 2), highlighting the intra-familiar phenotypic variability associated to the same mutation and suggesting a potential role of genetic, epigenetic or even environmental modifiers [49]. It is noteworthy to mention that the phenotypic variability in this family is most likely not age-dependent since the learning and developmental problems were very pronounced in the elder sibling (FB22: II.1) back from childhood, whereas his sister (FB22: II.2) was able of pursuing a higher level in school and has better social skills.

The *BBS12* gene mutated in FC51 encodes a chaperonin-like protein which is part of a chaperonin complex consisting of three BBS proteins: BBS6, BBS10, and BBS12 [46]. This complex is essential for the stabilization and formation of the BBSome core complex [46], initiating the first step of BBSome assembly and mediating the interaction with canonical chaperonins that perform the folding activity; indeed, no functional complexes are formed if this BBS-chaperonin complex is affected [50]. BBS12 is also involved in adipogenic pathways, pointing out to the obesity issue that is a major feature in BBS-patients [51]. The combination of two *BBS12* mutations was suspected to determine the BBS phenotype in FC51. In fact, the deleterious effect of the first mutation M3; p. (Leu89Valfs*11) will either result in a severely truncated protein missing 610 out of 710 amino acids, which is more than 85% of the protein’s total length, or lead to nonsense-mediated mRNA decay [52]. On the other hand, the second *BBS12* mutation M4; p. (Tyr294Asp) was never seen in any of the general populations included in different public sequencing projects. M4 replaces the neutral and uncharged Tyr294 residue with an acidic and negatively charged Asp residue. Furthermore, Tyr is a very large residue due to its aromatic group while Asp is small. All these chemical and structural alterations, in addition to the absence of the variant in common databases and its segregation with the disease, support its pathogenicity even though *in-silico* prediction tools did not predict the latter. Moreover, we detected a third variant (M5) in this patient, in another BBS gene (*BBS5*): the p. (Asn184Ser). Although triallelism was previously suspected in many BBS families [53,54,55,56,57,58], however, we could not consider this variant a disease-causing mutation related to a case of triallelic/digenic mode of inheritance, because such case needs the presence of three pathogenic mutations, which is not the situation of this family. Indeed, this mutation was present in a homozygous state in several general populations with a relatively high frequency (Table 3); inconsistent with its pathogenicity. Most importantly, this same variant was previously suspected to be a BBS modifier along with a homozygous *BBS1* mutation [38], but we were not able to consider it a phenotype modifier in our case due to the lack of other cases harboring the same *BBS12* mutations (M3 and M4) without the presence of the *BBS5* variant (M5). Indeed, the presence of such cases is needed to be compared with the case of index FC51: II.2, in order to firmly assess the exact contribution of variant M5.

Our last family (FD10) presented USH that was reflected by a homozygous mutation in *CLRN1*. Three clinical types of USH have been described in literature: USH type I (USH1), type 2 (USH2), and type 3 (USH3). USH1 is denoted by congenital severe-to-profound hearing loss, vestibular dysfunction, and an onset of RP within the first decade. USH2 is characterized by congenital moderate-to-severe hearing loss, absence of vestibular dysfunction, while RP manifests from the second decade onward. In USH3, patients present progressive RP, a late hearing loss that usually develops within the first two decades, as well as variable vestibular dysfunction [8,59].

*CLRN1* mutations are mainly associated with USH3 [60,61], which is consistent with the symptoms of FD10: III.3 who started experiencing vision problems at 15 years; however, her progressive hearing loss only began at the age of 28. Occasionally, she also suffered from dizziness and imbalance. *CLRN1* encodes the clarin-1 protein that is potentially involved in signaling, sorting of vesicles and development and/or maintenance of stereocilia [62]. The detected homozygous mutation M6; p. (Tyr63Ser) was not previously detected in any of the searched databases, not even in heterozygous state. It was predicted to be pathogenic by all prediction tools owing to the fact that it replaces the highly conserved Tyr63 which is hydrophobic, aromatic and very large by a Ser residue that is hydrophilic and very small.

The current study expanded the clinical and mutational spectra associated with BBS and USH. At the mutational level, only few studies targeting these diseases in the Lebanese population were published [63,64,65,66,67,68,69,70]. Regarding BBS, only three Lebanese BBS families were shown to carry pathogenic mutations in *BBS10, BBS2,* and *BBS8* [64,65,68,69]. In this study, other BBS genes (*BBS9,* *ARL6,* and *BBS12*) were found mutated for the first time in Lebanese patients. These mutations were likely pathogenic, reflecting the genetic and allelic heterogeneity of BBS in this population. On the other hand, four previous studies reported eleven USH Lebanese families [63,66,67,70]. *CLRN1*, which is mutated in our family FD10, was found mutated in only one of those eleven families [63].

In the last two decades, the introduction of high-throughput NGS technologies has enabled a tremendous progress in revealing the genes and mutations underlying IRD worldwide. Indeed, NGS allows a parallel massive screening of all related genes, improving both the efficiency and the cost of DNA sequencing [1,19], and increasing the diagnostic rate compared with conventional methods, as reported in several studies [12,71,72]. Genetic diagnosis is significantly relevant on many aspects, especially for familial genetic counseling, determination of recurrence risk in offspring, prenatal testing, and most importantly to select patients eligible for specific genetic therapies or trials [4].

## Figures and Tables

**Table 1:** Clinical results identified in five Lebanese patients with inherited retinal disorders.

**Family**	FA4	FB22	FB22	FC51	FD10
**Individual**	FA4: V.3	FB22: II.1	FB22: II.2	FC51: II.2	FD10: III.3
**Gender**	Female	Male	Female	Female	Female
**Disease**	BBS	BBS	BBS	BBS	USH
**Age**	24	34	28	16	40
**Age at Onset of RCD**	6	3	3	11	15
**Age at Diagnosis**	12	5	3	13	28
**Visual Acuity (O.D/O.S)**	20/30–20/200	Only HM—CF	20/400–20/400	20/100–20/125	20/30–20/40
**ERG**	Severely reduced photopic and scotopic ERG	Severely reduced photopic and scotopic ERG	Severely reduced photopic and scotopic ERG	Severely reduced photopic and scotopic ERG	Severely reduced photopic and scotopic ERG
**Fundus Photography**	Peripheral pigmentary changes associated with macular involvement	Bilateral widespread pigmentary changes outside the vascular arcades	Bilateral widespread atrophic changes outside the vascular arcades	Atrophy and reduced autofluorescence at the macula	Peripheral retinal pigment and central hyper-fluorescence
**Optical Coherence Tomography**	Bilateral retinal layer thinning at the macula	Reduction of retinal thickness	Bilateral retinal thinning	Bilateral thinning of the neuroretinal layers at the macula	NA
**Other Symptoms**	Table 2	Table 2	Table 2	Table 2	Hearing loss

BBS: Bardet–Biedl syndrome, RCD: rod cone dystrophy, OD: oculus dexter; OS; oculus sinister; HM: hand motion; CF: count finger; ERG: electroretinogram, NA: not available.

**Table 2:** Bardet–Biedl syndrome major and minor features in four affected individuals, listed according to the diagnostic criteria published by Beales et al. [10].

	FA4: V.3 (24 YEARS)	FB22: II.1 (34 YEARS)	FB22: II.2 (28 YEARS)	FC51: II.2 (13 YEARS)
*MAJOR FEATURES*				
Rod-cone dystrophy	+	+	+	+
Truncal obesity	+	+	+	+
Polydactyly	−	−	+	−
Genital anomalies	−	−	−	−
Renal anomalies	−	−	−	−
Learning difficulties	+	+	+	+
*MINOR FEATURES*		−		+
Speech disorder/delay	−	+	−	−
Development delay	+	+	+	−
Dental anomalies/hypodontia	+	+	−	+
Strabismus/cataracts/astigmatism	−	−	+	−
Diabetes mellitus	−	+	+	−
Brachydactyly	−	+	+	−
Syndactyly	−	+	+	+
Clinodactyly	−	+	+	−
Imbalance/coordination problems	+	−	+	−
Anosmia/hyposmia	−	−	−	−
Congenital heart defects	−	−	−	−
Hepatic fibrosis/Liver disease	+		−	

+: present; −: absent.

**Table 3:** Mutations identified in four Lebanese families with BBS and Usher syndrome (USH).

Family	Disease	Gene Reference Sequence	Exon	rs ID	Nucleotide Exchange	Amino Acid Change	Frequencies	PolyPhen-2	SIFT	Mutation Taster	Novel/Reported
								(Score)	(Score)	(Score)	
FA4	BBS	*BBS9*	20	rs61764068	c.2258A > T	p. (Glu753Val)	0.0007685 (ExAC)	Probably damaging (0.999)	D	Disease causing (0.998)	Reported by [36]
		NM_001348041.4					0.0007475 (gnomAD) 0.0006769 (TOPMed)		(<0.05)		
							Never Hom				
FB22	BBS	*ARL6*	3	rs1359075294	c.68T > C	p. (Leu23Pro)	0 (ExAC)	Probably damaging (0.999)	D	Disease causing (0.999)	Novel
		NM_032146.5					0 (gnomAD)		(<0.05)		
							0.0000079 (TOPMed)				
							Never Hom				
FC51	BBS	*BBS12*	3	rs1397714772	c.265_266delTT	p. (Leu89Valfs*11)	0 (ExAC)	−	−	Disease causing (1)	Novel
		NM_001178007.1					0.000012 (gnomAD)				
							0.0000079 (TOPMed)				
							Never Hom				
		*BBS12*	3	No rs	c.880T > G	p. (Tyr294Asp)	0 (ExAC)	Benign (0.022)	T	Polymorphism	Novel
		NM_001178007.1					0 (gnomAD)		(>0.05)	−0.999	
							0 (TOPMed)				
							Never Hom				
		*BBS5*	2	rs137853921	c.551A > G	p. (Asp184Ser)	0.004381 (ExAC)/1 Hom	Probably damaging (1)	D	Disease causing (0.999)	Reported by [37]
		NM_152384.3					0.00416 (gnomAD)/2 Hom		(<0.05)		
							0.0038 (TOPMed)/2 Hom				
FD10	USH	*CLRN1*	1	No rs	c.188A > C	p. (Tyr63Ser)	0 (ExAC)	Probably damaging (1)	D	Disease causing (0.999)	Novel
		NM_001195794.1					0 (gnomAD)		(<0.05)		
							0 (TOPMed)				
							Never Hom				

HOM: Homozygous; D: Deleterious; T: Tolerate.

## Supplementary Materials

The following are available online at https://www.mdpi.com/2073-4425/10/12/1047/s1 Table S1: Criteria used to classify sequence variants according to ACMG guidelines [36]. Table S2: Incidental findings detected by NGS in inherited retinal disorders genes other than Bradet Biedl and Usher syndromes.
