# Supplementary material for: Next Generation Sequencing Identifies Five Novel Mutations in Lebanese Patients with Bardet–Biedl and Usher Syndromes
Source: Genes (Basel). 2019 Dec 16;10(12):1047. doi: 10.3390/genes10121047 (PMC6947157; doi:10.3390/genes10121047)
Supplement: Supplementary file 1 [file genes-10-01047-s001.pdf]

Table S1. Criteria used to classify sequence variants according to ACMG guidelines (46).

**Variant ID**	**Evidences of pathogenicity**	**Class**
M1	1 moderate evidence (PM2) and 4 supporting evidences (PP1-PP2-PP3-PP4)	Likely pathogenic
M2	1 moderate evidence (PM2) and 4 supporting evidences (PP1-PP2-PP3-PP4)	Likely pathogenic
M3	1 very strong evidence (PVS1), 1 moderate evidence (PM2) and 3 supporting evidences (PP1-PP3-PP4)	Pathogenic
M4	2 moderate evidences (PM2-PM3) and 3 supporting evidences (PP1-PP2-PP4)	Likely pathogenic
M5	4 supporting evidences (PP1-PP2-PP3-PP4)	Uncertain significance
M6	1 moderate evidence (PM2) and 4 supporting evidences (PP1-PP2-PP3-PP4)	Likely pathogenic

M: mutation.

Table S2: Incidental findings detected by NGS in inherited retinal disorders genes other than Bradet Biedl and Usher syndromes.

Index Patient (corresponding disease)	Gene	Associated IRD	Status	Exon	rs ID	Nucleotide Exchange	Amino Acid Change	Frequencies	PolyPhen-2	SIFT
**FA4: V.3 (BBS)**	*CDH23* NM_022124.5	USH	HTZ	67	rs763993100	c.9587_9589delACA	p.Asn3197del	0.0000643 (gnomAD) 0.0000159 (TOPMed) Never Hom	-	-
	IFT140 NM_014714.4	RP or LCA	HTZ	20	rs200876696	c.2569G>A	p.Gly857Ser	0.003545 (gnomAD)/8HOM 0.001911 (TOPMed)/1HOM	Probably damaging	D
	RBP3 NM_002900.3	RP	HTZ	1	rs144289912	c.1795A>G	p.Ile599Val	0.001144 (gnomAD) 0.0009795 (TOPMed) Never Hom	Benign	D
	CDHR1 NM_033100.4	CRD	HTZ	8	rs747388076	c.764T>A	p.Val255Glu	0.00002386 (gnomAD) 0 (TOPMed)	Possibly damaging	D

									Never Hom		
**FB22: II.1**<br><br>**(BBS)**	*HGSNAT*<br><br>NM_152419.3	RP	HTZ	1	-	c.11C>A		p.Ala4Glu	0 (gnomAD)<br><br>0 (TOPMed)<br><br>Never Hom	Benign	-
	EYS<br><br>NM_001292009.1	RP	HTZ	4	-	c.476G>C	p.Cys159Ser	p.Cys159Ser	0 (gnomAD)<br><br>0.00000796 (TOPMed)<br><br>Never Hom	Probably damaging	D
	CDH23<br><br>NM_022124.5	USH	HTZ	68	rs200124827	c.9928C>T		p.Arg3310Cys	0.0002158 (gnomAD)<br><br>0.0001832 (TOPMed)	Possibly damaging	D (low confidence)
**FD10: III.3**<br><br>**(USH)**	*GUCY2D*<br><br>NM_000180.4	LCA	HTZ	4	rs140638938	c.1315G>A		p.Gly439Arg	0.000334 (gnomAD)//1Hom<br><br>0.0000079 (TOPMed)	Probably damaging	D
	GUCY2D<br><br>NM_000180.4	LCA	HTZ	20	rs552184470	c.124_129delCTGCTT		p.Leu44_Leu45del	0.001913 (gnomAD) /3Hom	-	-

							0.002087 (TOPMed) /3Hom		
PDE6A NM_000440.3	RP	HTZ	14	rs765007196	c.1730C>T	p.Thr577Met	0.000007962 (gnomAD) 0.0000079 (TOPMed) Never Hom	Possibly damaging	D
RGS9 NM_003835.4	recessive delayed cone adaptation	HTZ	5	rs201997888	c.314C>G	p.Thr105Arg	0.001901 (gnomAD)//1Hom 0.001776 (TOPMed)	Probably damaging	D

HTZ: heterozygous; HOM: homozygous; D: deleterious, USH: Usher syndrome; RP: retinitis pigmentosa; LCA: Leber's congenital amaurosis; CRD: cone rod dystrophy.
